# Using Implementation Research to Inform Scaling of Parenting Programs: Independently Conducted Case Studies from Zambia and Bhutan

**DOI:** 10.3390/children11040477

**Published:** 2024-04-16

**Authors:** Frances Aboud, Karma Choden, Given Hapunda, Francis Sichimba, Ania Chaluda, Rafael Contreras Gomez, Rachel Hatch, Sara Dang, Karma Dyenka, Cecilia Banda, Carina Omoeva

**Affiliations:** 1 Department of Psychology, McGill University, Montreal, QC H3A 1G1, Canada; 2 Independent Researcher, Thimphu 22001, Bhutan; karmacdn@gmail.com; 3 Department of Psychology, Great East Road, University of Zambia, Lusaka 10101, Zambia; given.hapunda@unza.zm (G.H.); francis.sichimba@unza.zm (F.S.); 4 FHI 360, 2101 L Street NW, Washington, DC 20037, USA; achaluda@fhi360.org (A.C.); rcontrerasgomez@fhi360.org (R.C.G.); rhatch@fhi360.org (R.H.); comoeva@fhi360.org (C.O.); 5 Save the Children, 899 North Capitol Street NE, Suite 900, Washington, DC 20002, USA; sdang@savechildren.org (S.D.); karma.dyenka@savethechildren.org (K.D.); 6 UNICEF Zambia, UN House, Alick Nkhata Road, P.O. Box 33610, Lusaka 10101, Zambia; cbanda@unicef.org

**Keywords:** parenting, implementation research, low- and middle-income countries, scaling, Bhutan, Zambia

## Abstract

Two case studies of parenting programs for parents of children 0 to 36 months of age, developed and implemented by Save the Children/Ministry of Health/Khesar Gyalpo University in Bhutan and UNICEF Zambia, were conducted by an independent research group. The focus was on how program delivery and scale-up were revised on the basis of feedback from implementation research. Feedback on workforce delivery quality was based on observations of deliveries using a monitoring form, as well as survey and interview data collected from the workforce. In-depth interviews with the resource team during the fourth year of implementation revealed how the feedback was used to address horizontal and vertical scaling. Delivery quality was improved in some cases by revising the delivery manual, offering refresher courses, and instituting regular monitoring. Scaling challenges in Zambia included slow progress with regard to reaching families in the two districts, which they addressed by trialing group sessions, and stemming workforce attrition. The challenges in Bhutan were low attendance and reducing the workload of providers. Vertical scaling challenges for both countries concerned maintaining demand through continuous advocacy at community and government levels to sustain financing and to show effectiveness in outcomes.

## 1. Introduction

Parenting programs for parents of children from birth to 36 months of age are aimed at helping caregivers provide the responsive stimulation that children need to reach developmental milestones [1]. A recent meta-analysis of parenting programs found, on average, moderate effect sizes in improving both parenting practices and child developmental outcomes [2]. Based on this evidence, the World Health Organization (WHO) published its Guidelines for Improving Early Childhood development [3] and the Nurturing Care Framework [4], both emphasizing the need for caregivers to provide their child with stimulation and communication in a responsive manner. Ministers of Health worldwide responded with interest to the call and agreed to promote early child development in their countries through the provision of parenting services.

In this paper, we describe two parenting programs, one in Zambia and a second one in Bhutan, and their efforts to scale both horizontally within the country and vertically through the governmental health system. We use the ExpandNet definition of scaling: “deliberate efforts to increase the impact of innovations successfully tested in pilot or experimental projects so as to benefit more people and to foster policy and program development on a lasting basis” [5]. The phrase “benefiting more people” reflects activities aimed at scaling horizontally or geographically, while the phrase “foster policy and program development on a lasting basis” reflects a vertical or system level scale. Both programs were in their fourth year of transitioning to scale when feedback data on the quality of service delivery was provided by FHI 360, serving as an independent implementation research-and-learning partner to the funding agency. Subsequent efforts to engage in program development to address evidence of challenges were then tracked. We start by describing the two parenting programs in terms of their curriculum, dosage, and workforce. We then review the background literature on scaling parenting programs and the use of implementation research to identify and manage challenges.

### 1.1. Programmatic Features 

The two parenting programs presented here are Care for Child’s Healthy Growth and Development (CCD, or Nurturing Care for Early Childhood Development) implemented by UNICEF and the Ministry of Health in Zambia, and Prescription to Play (P2P) implemented by Save the Children, Ministry of Health and Khesar Gyalpo University of Medical Sciences in Bhutan (and based on their Building Brains Common Approach). Both were funded by the LEGO Foundation and began scaling their programs in 2020. The UNICEF CCD program was evaluated on several occasions in other countries with small or mixed effects on parents and children [6,7] but was never piloted in Zambia. The Save the Children P2P program was evaluated as a pilot in Bhutan with positive effects [8].

The design of a program, its curriculum, mode of delivery, and intensity are important to consider [9,10,11]. They have a bearing on whether the program will be successfully implemented, given the workforce and the beneficiaries. For example, the intensity or dosage of contacts with parents is critical: it may need to be higher for parents who are not yet engaging in responsive stimulation. The inclusion of behavior change strategies built around active learning may be necessary [12]. These strategies include showing parents a new game to play with their child using home-available materials, such as cloth, cups, and sticks, letting parents practice playing the game with their child during the session and offering positive and constructive feedback to the parent. The same procedure is carried out when showing parents how to use responsive talk about a picture. Features of these two programs indicate a number of differences and some similarities. Funding from the LEGO Foundation was related to the inclusion of play and nurturing care in their curriculum. So, both focused on play and communication, with additional messages about nutrition and hygiene. 

Earlier publications, confirmed by our current research, showed that Zambian rural parents (here, the term “parent” is used to include any “primary caregiver”) had relatively low levels of responsive stimulation, averaging 26 or 27 out of 45 on the HOME Inventory measure; parent education averaged around 5 and 6 years of schooling [13,14]. Home visits to parents using CCD occurred seven times over a period of 24 months or six times over 12 months. Community-based volunteers who delivered the program to parents were likewise not experienced in early child development and responsive stimulation [data for a recently trained cohort were collected and presented as part of this study]. Training was initially outsourced to non-governmental organizations and then transferred to the Ministry of Health and Ministry of Education district officers. Each visit made use of the CCD flipchart with illustrations of play and a one-page outline of what to say and ask. Each visit also included messages about nutrition and illness. According to initial plans, the goal was to train 670 community-based volunteers (CBVs) and scale up in two rural districts in the Eastern Province with a reach of 50,000 families. In some communities, a physical ”hub” was built to house activities related to growth monitoring and stimulation sessions. Regarding vertical scale, they aimed to train health workers in 67 target clinics and train district health officers to provide supervision. UNICEF worked closely with the Ministry of Health and its multi-ministry committee. Multi-sectoral committees were also established at the district levels.

For the Prescription to Play parenting program in Bhutan, parents were recruited from mainly rural families registered with the nearest Primary Health Center or Outreach Clinic for their child’s basic health care. Current research found that levels of responsive stimulation of districts before their inclusion in the program averaged 30.5 out of 45 on the HOME Inventory [13]. Mothers’ education averaged 7 to 8 years of schooling. Twelve group sessions lasting 60–90 min were conducted monthly with anywhere from 5 to 50 mothers and children. Of these 12, 9 emphasized play and communication such that 4 games for different age groups were demonstrated and practiced at each session. Health assistants who delivered these group sessions were considered professionals and part of the health system, but they were initially unfamiliar with early child development. They had responsibilities at the clinic in addition to parenting sessions. The goal of Prescription to Play was to train 640 health assistants (HAs) working in 264 clinics and 551 outreach sites and to scale up in three phases across the country’s 20 districts. Training of health assistants was institutionalized within the Khesar Gyalpo University of Medical Sciences. Regarding the process of vertical scale, the organization met initially and regularly with a focal division from the Ministry of Health who found the program compatible with their policy on “1000 Golden Days”.

### 1.2. Implementation Research on Scale-Up

Very few parenting programs have actually been scaled-up and sustained, though several show features of transitioning to scale. ExpandNet’s definition of scaling [5], described previously, includes geographic coverage, although the reach has not been explicitly defined in terms of beneficiaries or geography. Likewise, the definition includes vertical expansion that facilitates sustaining the program, for example, by developing a policy framework within the government and integrating the workforce within the health care system, including training and supervision. Three key elements are included in the ExpandNet framework, starting with the Innovation (the parenting program); the Resource Team initially implementing the program (here, UNICEF in Zambia and Save the Children in Bhutan); and the User Organization—in this case, the governments’ Ministry of Health. Data for the two case studies reported here come from the past 18 months during the third and fourth year of implementation when both programs were in transition to scale, specifically conducting activities to improve implementation of the program throughout the health system by expanding their reach and transferring the program to the government.

How monitoring and evaluation is used by the Resource Team (also called the implementing partner) to improve the program is one important scaling strategy identified by ExpandNet [5]. A scoping review of scalable parenting programs identified the monitoring system as a critical component of scaling programs [10]. It includes the use of data collection tools to track program activities and the use of monitoring findings to make data-informed decisions. Decisions commonly address the training and supervision of delivery agents in order to maintain and improve quality. Decisions might also make rapid modifications of the curriculum and its mode of delivery. An extensive analysis of Criança Feliz, the CCD scaled-up program across Brazil’s municipalities, identified positive and negative feedback loops as relevant for such informed decision making [7]. 

Implementation research for the current study was conducted by an independent research organization, with results and interpretations fed back to the implementing partner shortly after analysis as part of monthly meetings. The independent research partner, FHI 360, was engaged and funded by the LEGO Foundation to collect data on the implementation process of each program as they began to scale up. This was conducted through a collaborative arrangement with the implementing partners (UNICEF, Save the Children) so that there would be no duplication of data collection. For the purposes of this paper, we present data on the quality of delivery by service providers during the first and third years of operation. Delivery quality is important to maintain as a program progresses towards scale [15]. It is essentially the linchpin of a parenting program—the point of contact between the program and the beneficiaries. Evidence points to the association between the quality of delivery and subsequent improvements in parents’ practices [16]. How this information was used to inform changes to the program—its curriculum, mode of delivery, training and supervision of the workforce, and advocacy with the government—was documented from surveys and interviews with two cohorts of providers and a resource team.

Feedback also came from the workforce, some of whom were interviewed a year prior. A great deal was written about supervision and the need for two-way feedback from the supervisor to the service provider and from the provider to the supervisor [17]. A cohort of service providers participated in in-depth interviews both during Round 1 assessments in 2022 and again in 2023. They were asked to comment again on the same challenges to scale raised in the year prior. This information was fed back to the resource team to determine how they would address workforce and delivery challenges.

### 1.3. Research Objectives 

Both programs were in their third and fourth years of transitioning to scale when feedback data on the quality of service delivery was provided by the independent research partner. Subsequent efforts by the workforce and resource team to address findings were then described, according to each perspective.

The overall objective was to describe findings on the quality of delivery by the workforce of the two parenting programs, along with subsequent actions taken to address limitations. Based on both quantitative delivery observations and qualitative interview data, the specific objectives were as follows:

1. Evaluate the quantitative findings concerning the quality of delivery as monitored over two years while the programs scaled up.
2. Describe the subsequent perspectives and actions of the workforce as they relate to the feedback on delivery quality.
3. Describe the actions reported by the resource team as they addressed feedback on delivery quality and describe the critical challenges to scaling both horizontally across geographies and vertically through the system.

## 2. Materials and Methods

Previously used observational methods for assessing workforce delivery quality were applied along with survey and in-depth interview methods [18]. The CARE reporting guidelines for the implementation of early childhood development programs were used [19]. Ethics approval was obtained from ethics committees at FHI 360, McGill University, and Ethics Review Boards in Zambia and Bhutan. Anonymity and confidentiality of the data from both workers and the resource team were maintained as much as possible despite the small sample size in some cases.

### 2.1. Participants 

Participants comprised frontline workers (also referred to as providers) who delivered the parenting program directly to parents and their children, namely the community-based volunteers (CBVs or volunteers) in Zambia and Health Assistants in Bhutan. As in most East African countries, the Zambian program found that unpaid volunteers, possibly already delivering other community nutrition/health programs, were more available than professionals and less costly. The Bhutanese program initially tried utilizing village health workers but found they did not have the skills required and were not as respected as professional workers. Two rounds of observations were made of 60 different randomly selected providers in each country as they conducted home visits or group sessions. Workers for the phone survey were chosen at random from the pool of providers who, after recent training, had delivered the program for several months. Sample size was based on an expected improvement of 5 points out of 40 (1 SD) in the knowledge test over time, leading to a required sample of 128. A purposive subsample of previously trained workers, who varied in gender and educational background, was selected to participate in annual in-depth interviews. Here, we include their second round of interviews from 2023. Saturation was expected to be reached with ten participants; due to attrition in the Bhutanese sample (moving to a new position or further education), their numbers were fewer. A second group of participants comprised members of the resource teams who were responsible for providing technical support to the programs. Informed consent was obtained from each participant at the time they were observed or interviewed.

### 2.2. Measures and Procedures

Table 1 outlines the four methods used to gather information. Three of these four methods include aspects of workforce engagement, including observation of their delivery and perspectives on their delivery and support. The fourth method comprises in-depth interviews with members of the resource team to identify how they addressed the implementation feedback and met challenges to scale. Sample sizes and dates of data collection are included. Details on each measure and data collection procedure follow. Finally, the procedure for providing implementation feedback to the implementing partner is described. Copies of measures may be requested from the authors.

#### 2.2.1. Delivery Observations 

Based on previously published measures of delivery quality [16,18], qualities found to be important in changing parenting practices are listed. Ten qualities concern specific activities related to active teaching/learning, known as behavior change techniques [12]. They include introducing a new play or communication activity, demonstrating the activity, giving parents an opportunity to practice the new activity with their child, and coaching them on the new practice. If not observed, the item is scored 1; if observed but delivered in a cursory, confusing, or inadequate manner, it is scored 2; if observed and delivered in a very good manner, it is scored 3. Four additional items concern qualities that can be observed throughout the session: if the provider appears to be prepared, covers the content of the session, delivers it in an engaging manner, and expresses acceptance and empathy towards parents. These are scored 1 (poor), 2 (inadequate, could be improved), and 3 (very good). Three qualities of parent participation are noted as yes or no for home visits and as few (25%), some (50%), or many (75%) for group sessions: parents report engaging in the new practices since the last contact (homework); parents practice activities with their child during the session; parents have their own play objects available during the session. For training purposes, a more detailed description is provided for each quality.

Training of observers from an independent local data collection firm was conducted over three or four 3 h sessions using written vignettes of hypothetical parenting sessions based on the program being delivered (CCD for Zambia, P2P for Bhutan). These young adult males and females had post-secondary education and were hired and paid per job. There was only some overlap between those who conducted two delivery observations. Trainees were required individually to rate every instance they saw in the vignette that reflected one of the qualities to be observed. Over the course of three separate assignments, inter-rater reliability with the trainer reached 80% and above for all observers. 

#### 2.2.2. Phone Survey of Workforce 

The structured survey consisted of 20 questions with sub-questions [18]. It included demographic variables (age, sex, years of education, years of experience); workload for this program and for other duties; training and supervision; confidence in delivery; perspective on own work; and the 20-item Caregiver Knowledge of Child Development (CKCD) Inventory with 10 items on child development and 10 items on stimulating parenting practices (Ertem, 2007) [20]. Because these cohorts were recently (past year) trained to deliver the parenting program and as their service was intended to extend the reach of the program, we focused on their workload and their training and knowledge of child development and parenting. Enumerators at a local data collection firm in each country, similar to those described in Section 2.2.1, were hired and trained to deliver the survey by phone. Up to three attempts were made to reach the interviewee; if this was unsuccessful, an alternative person on the list was recruited. Answers were recorded on the Kobo Collect application and analyzed using descriptive statistics. A second enumerator repeated 10% of the surveys 2 or 3 weeks later to assess consistency.

#### 2.2.3. In-Depth Interviews with Workforce 

The semi-structured interview guide requests detailed information on providers’ understanding of the program they deliver and the parents who attend. Questions are open-ended. Items include methods used in their own training; supervision and peer support; workers’ understanding of the terms “early child development” and “responsive stimulation”; and challenges they encounter in their work. Because the interviewees comprised a longitudinal cohort that was interviewed the previous year, we focused on questions and comments that concerned changes to the program, their delivery, and their training. Local researchers who were part of the research team conducted the interviews by phone or in person. Each interview took approximately 40 to 60 min to complete. The interviews were audio-recorded, transcribed, and translated into English. Both the local researcher and the lead international researcher read through the transcripts and conducted a content analysis, following the sequence of interview questions and probes. The local researcher first produced a summary of the key responses to each question and representative quotes. The international researcher, after reading the interviews translated to English, added content and sometimes quotes. Differences in emphasis were resolved between the two coders. Before summarizing the findings for this paper, the international researcher re-read the transcripts and highlighted representative quotes that addressed changes to the program. The identifying features of those quoted are not provided because, with a small sample, to do so might jeopardize anonymity.

#### 2.2.4. In-Depth Interviews with Technical Persons from the Resource Team

The semi-structured interview guide focuses on activities and challenges relating to scaling the parenting program horizontally and vertically, based partly on the ExpandNet materials [5]. This includes how technical persons were modifying the program and/or its delivery based on feedback from implementation research and how they were addressing the critical challenges to scaling the program. Questions were open-ended. A local researcher and an international researcher conducted the interview during July and August 2023. The interview was transcribed and subjected to content analysis in order to answer the research questions. The analysis and selection of representative quotes followed a sequence similar to what was described for workforce interviews, focusing on how the resource team used feedback to scale. Again, the features of those who were quoted was not provided because, with this small sample, it would jeopardize anonymity.

#### 2.2.5. Feedback Procedure from Implementation Research Team to Resource Team

Feedback from the implementation research-and-learning team to the resource team in each country took three forms. First, feedback was provided orally on Zoom during monthly or bimonthly meetings starting in 2021 and continuing throughout the course of the project. At this time, whatever data were recently analyzed from workforce delivery and interviews were presented as a slide deck, including the method and findings, along with time for comments and questions. Comparison data from previous rounds were also presented and the changes were discussed. Local researchers sometimes offered context-specific recommendations. Secondly, feedback took the form of written reports comprising 10–15 pages that was sent to the resource team with the methodology, results in tabular and text form, comparisons with previous findings, and several bulleted conclusions. This would occur 2 to 3 months after the oral presentation in order to consider newly offered interpretations. These two feedback procedures therefore occurred within six months of data collection. The third occasion for feedback was an in-person meeting, including the research and resource teams, over three days in November 2022 when the findings from all implementing programs over the previous years were presented and discussed face-to-face in the presence of all resource teams. Those who requested help in addressing delivery quality findings and other problems had time to do so with the research team and other resource teams. Seven to ten months after this meeting, the most recent data from 2023 were collected.

## 3. Results

Results for the four methods of data collection are presented in descriptive form. Data were collected by independent enumerators and researchers in each country, and all analyses were conducted by independent analysts.

### 3.1. Delivery Observations 

Delivery quality on ten activities by community-based volunteers in Zambia was scored a mean of 1.7 out of 3 in the first round and 1.8 in the second round—20 months later, with another cohort of providers (see Table 2). Asking about homework improved substantially, with 68% of providers applying this action very well; other qualities showed small declines, and the use of visual aids, namely the counselling cards, declined sharply. Counselling cards are the key job aid used by providers to present content on play and communication to parents. In terms of style, the delivery skills of this cohort also declined, especially in terms of being engaging and showing acceptance and empathy towards parents. More parents claimed to have practiced between home visits, and more of them practiced with their child during the visit; however, only a third had their own play materials at the home visits.

The ten quality activities delivered by health assistants in Bhutan received a quality score of 2.4 out of 3 on Round 1 and 2.7 on Round 2 (see Table 3). They showed significant improvements on all items, particularly asking about homework (playing the new games with their child between sessions), coaching on responsiveness, demonstrating a new practice, giving parents the opportunity to try it with their child, and coaching. Their delivery skills also improved, especially in terms of being more lively and engaging with parents. In contrast to Round 1, most parents in Round 2 were seen to be engaged in playing with their child during the session.

### 3.2. Survey of the Workforce

The workforce in Zambia comprised community-based volunteers with an average of 10 years of education and 9 years in their position as a volunteer (see Table 4). They had worked on the current Care for Child Development program for 6 months and were trained over 6 days to deliver the nutrition, health, and development messages. They had contact with 3.8 families over the week prior to the survey (though they reported 10 families in their caseload). Most had other responsibilities for 5 h per week, perhaps working as a volunteer for other programs or working to make money, but most did not feel overworked. They were confident of their own delivery, received administrative supervision, and met frequently for peer support; none reported onsite supervision. Their knowledge of child development and parenting stimulation was minimal according to the CKCD Inventory test taken at this time. As usual, providers often made the mistake of assuming that stimulating practices should be offered at older ages, thus depriving children of their needed stimulation in infancy.

The workforce in Bhutan comprised professionals with, on average, 13 years of education and almost 16 years in their positions as health assistants. They worked on the current Prescription to Play program for 7.4 months and were trained over 11 days to mainly deliver the child development messages along with some messages on safety, security, nutrition, and hygiene; most had received a refresher course, although few were supervised. They had contact with, on average, 10 families per group per month. All had other responsibilities, such as clinical work, averaging 37.2 h per week. They became more confident of their own delivery with experience. Their knowledge of child development and parenting stimulation was minimal according to the Caregiver Knowledge of Child Development.

### 3.3. In-Depth Interviews with Workforce

Data from the two countries are presented separately in the form of quotes. This feedback, given to the resource team in the form of oral presentations and written reports, was both positive and negative. For example, providers in both countries reported appreciating demonstrations and role-playing practice during their initial training. They also benefited from the support they received from peers.

Volunteers in Zambia, interviewed for the second time in mid-2023, claimed that though the counselling cards were critical for delivering home visits, they had not been revised to include any more behavior change techniques and the cards were not translated into the local language (see Table 5). Although volunteers knew that songs would be an appreciated form of communicating with children, none were added to the manual. Supervision was mainly administrative, namely handing in reports of who was visited and when. No refresher training was received by this cohort despite over 18 months of delivering the program. Peer support was deemed regular and helpful. One expressed a strong desire to be monitored/observed by peers while delivering a visit. Concepts like early child development and responsive stimulation, assessed after training and during the phone survey using the CKCD Inventory, were still only vaguely understood.

Many volunteers commented that the six visits over 12 months (seven over 24 months) were insufficient to change parents’ practices. Parents were not playing in between visits and appeared not to grasp how to play or communicate with their child. Volunteers were therefore visiting more frequently and repeating stories and questions from the same counselling card on several visits. Some families did not give priority to the visits; the Headman helped to sensitize the community to the importance of the parenting program. Volunteers asked for t-shirts identifying their status, along with a mode of transport, and remuneration for their work.

In Bhutan, health assistants, interviewed for the second time in mid-2023, appreciated the revised version of their manual with updated instructions on the delivery qualities they had previously lacked (see Table 6). They relied on it to guide their delivery. This cohort claimed to have more peer support than before, but their supervision was mainly administrative. They claimed to have discussed the challenge of poor attendance during a PDSA (plan-do-study-act) session and had made their own plans on how to address it, but were skeptical about solving the problem. A refresher was offered but some could not attend due to conflicts. Most were clear on the concept of early child development as related to brain and mental maturity and understood the concept of stimulation but were confused about the meaning of “responsiveness”.

There were mixed reactions to the question as to whether monthly sessions were sufficient. Some thought they were sufficient, and others preferred sessions being carried out twice monthly but knew it was not feasible given their clinic schedule. They were aware that only about 50% of parents were playing and communicating with their child between sessions. The two main challenges were poor attendance by parents at sessions and health assistants’ heavy workload. Some offered suggestions for where to find additional group facilitators.

### 3.4. In-Depth Interviews with Technical Support from the Resource Team

Results from the two country resource teams were organized into three sections (Table 7). The first included their responses to the delivery observation feedback from the implementation research team. The second included their responses to feedback concerning horizontal scaling, and the third included their responses to challenges about vertical scaling. In Zambia, the horizontal challenge was the current limited reach of the program in the two designated districts, and the vertical challenge was expanding and institutionalizing workforce training and supervision, and raising and sustaining demand at the government level and the community level through advocacy. In Bhutan, the horizontal challenge was to increase the attendance of registered parents at group sessions, and the vertical challenge was to advocate with the government to adopt the core of their program.

The resource team for Zambia’s CCD disseminated implementation findings to stakeholders and facility supervisors, but no changes were reportedly made to the counselling cards, training, refresher courses, or supervision; translation of materials was ongoing. Supervision by peers was being considered.

Reducing the number of visits to families in their caseload from ten to seven was being considered as a way of extending horizontal reach. Reach was to be extended by initiating group sessions in communities; the outcome of this plan is not known. Light-touch messages at the health facilities and mass media were created and disseminated; the effect of this exposure was to be studied. 

Vertical challenges to expand and institutionalize the workforce were met by training pre-service nurses on the curriculum and by training a cohort of master trainers who could continue training the frontline workforce. Government regulations regarding the registration and payment to volunteers were being applied. In addition, advocacy with the Ministries of Health, of Finance, and of Local Government were ongoing to create a policy, to provide output indicators for financing, and to work with the district government to whom decisions were now devolving. Output indicators were also integrated into the national information system, but monitoring and evaluation remained a challenge. They were optimistic about the demand for the program being present at the local level, though it needed to be renewed through contact with community Headmen.

The resource team for Bhutan’s P2P program instituted a number of changes to their manual and to training that addressed implementation research feedback on lower-scoring delivery items (see Table 8). They revised sections concerned with giving more emphasis to home practice, problem solving, responsive play, and to practice and coaching. Because providers rely so heavily on the manual when preparing and delivering the sessions, revisions to the manual were key. They tried several means to conduct monitoring of the providers during delivery and finally settled on a temporary solution, namely to hire temporarily a local NGO to provide current feedback.

Challenges to horizontal scaling include increasing the attendance of those registered for sessions; all districts now had group sessions, so the main problem was attendance. Several solutions were tried , including shortening sessions, making sessions more attractive, letting providers offer suggestions, and encouraging community mobilization. The team seemed to be aware that not all of these solutions would work smoothly. Challenges to vertical scaling came mainly from within the government as the focal division in the Ministry of Health had recently changed: concerns included the effectiveness of the program, its cost, and the additional workload it placed on health assistants. The resource team were brainstorming solutions to these problems.

## 4. Discussion

The findings revealed how the two programs addressed challenges to scaling up in three areas, specifically how they used information about the quality of providers’ delivery, about horizontal scaling, and about vertical scaling through the system. The P2P program in Bhutan took specific steps to improve the quality of delivery with changes to their manual, by hosting extra refresher courses, and by implementing a monitoring process. The CCD UNICEF team in Zambia planned to reduce the number of scheduled visits to families from ten to seven, but this might work against promoting a change in parental practices. Concerning horizontal scaling, the team in Zambia added group sessions to include more families as well as many light-touch media communications. The P2P Save the Children team in Bhutan expanded into 15 more districts and was trying to solve problems with community mobilization and attendance at sessions. Concerning vertical scaling, both teams conducted continuous advocacy at the community and the government levels and worked to sustain financing and to show effectiveness of their programs. CCD in Zambia additionally tried to develop strategies to maintain training of their frontline workforce and incentivize them through a government registry. P2P in Bhutan established a sustainable training partner at the university for pre-service training, but the in-service workforce was overburdened and the new ministry division had questions about cost and workload. 

The discussion will be organized around three themes: The first theme interprets different ways that delivery quality feedback was used to revise and improve programs as revealed in workforce survey and interview data. The second interprets ways that workforce feedback was used by the resource team to address horizontal and vertical scaling issues. The third examines the pros and cons of using a top-down (Zambia’s program) versus a bottom-up (Bhutan’s program) approach to scaling and sustaining a parenting program. 

### 4.1. Feedback Loops: Using Delivery Quality Feedback to Improve the Program

In Zambia, delivery quality did not show change over two annual rounds. Quality remained low. This was particularly the case for the ten qualities concerned with techniques of behavior change. The community-based volunteers frequently claimed that parents were not adopting the new play and communication practices, requiring that they return more frequently to speak to parents. The expected dosage of ten visits to discuss play and communication, which will possibly be reduced to seven, was apparently already too low to result in a change. The most recently trained volunteers who were implementing the programs rated their quality highly, presumably because they were doing what they were trained to do. However, the cohort who were interviewed yearly said there were no changes over the previous year in supervision or in the materials: they had not received a refresher course, most of their supportive supervision came from peers, and the counselling cards had still not been translated into the local language. No change was made to the content of the counselling cards that they depended on to guide what they said and did with parents, possibly because the Ministry was prioritizing a group counselling manual. Thus, the problematic issues related to delivery quality were not addressed since Round 1. The positive feedback from providers emphasized that peer supervision was frequent and very helpful, and that they often repeated a counselling card within the month if parents were not understanding and adopting the play practices. These extra responsibilities voluntarily undertaken to increase dosage and conduct peer supervision could be formalized and supported by UNICEF. 

In Bhutan, delivery quality improved considerably after feedback on Round 1. The group session providers raised the quality of many behavior change items, including asking about home practice, coaching responsiveness, giving parents the opportunity to practice the new games, coaching them on it, and helping to solve problems. The newly trained cohort of providers were very likely to have attended an in-person refresher course and this may have increased their level of confidence about delivery, despite few having received supervision. They were being monitored by an independent local organization using a measure based on the implementing researchers’ quality items. Interestingly, the Round 1 cohort who were interviewed again in Year 4 mostly claimed to have not received a refresher, though one was offered, and stated that they benefited from peer support only. They said that a newly revised manual was given to them and that it was much better; they relied heavily on their manual to conduct the 7-step sessions. 

### 4.2. Feedback Loops: Using Workforce Feedback to Inform Scaling

Challenges raised by the workforce pointed to issues that concerned horizontal and vertical scaling. Qualitative interviews with the resource team showed how they used feedback from the workforce to address these scaling challenges. The workforce in both sites claimed that demand for the program might be high among some parents but needed reinforcing among community leaders. The resource teams in both sites were very engaged in advocacy with the government in order to maintain Ministry stakeholders’ interest in sustaining the program through policy and budget decisions.

The major challenges raised by the workforce in Zambia included reach and lack of volunteers’ remuneration, leading to attrition. The resource team realized that only 20% of the expected beneficiaries in the two districts were visited with the current approach. Although volunteers said that ten home visits were insufficient, the plan to expand reach would result in them reducing their number of visits to seven. Training had been outsourced to local NGOs and then transferred to district health officers. Health workers at clinics and nurses were being trained but their contacts with families would be light-touch and opportunistic. Likewise, mass media efforts would be light-touch. If the parents receiving home visits had difficulties practicing play messages, then light-touch messages might not lead to improved practices either, though they might enhance community readiness to adopt new norms. The resource team developed a counselling manual for group sessions and hoped that this would expand reach in communities. Connected to this issue of reach was attrition of the volunteer workforce, who received no remuneration. The government produced guidelines for remuneration, but the process of registering volunteers and deciding on their remuneration took some time.

The two major challenges for the team in Bhutan were attendance and the heavy caseload of providers who had clinic responsibilities in addition to delivering group sessions. Attendance was mentioned previously by the yearly interviewed cohort. The resource team apparently had discussions with the workforce, providing them with suggestions and asking them to come up with their own solutions. Some providers were skeptical and demotivated, feeling that they had already made efforts to remind parents, but the resource team wanted them to make sessions shorter, livelier, and more appealing. Data on attendance were to be regularly collected to monitor the changes closely. The heavy caseload of health assistants conducting group sessions in addition to their clinic work required immediate action according to the government and the workforce. The resource team was helping in streamlining their group sessions, i.e., in shortening and making them more efficient. Another source of workers was being considered, namely facilitators who deliver preschool programs for children starting at 3 years of age. Although they are not as respected as health workers, they could provide supplementary assistance.

In brief, it appeared that resource personnel in both countries were responding to critical concerns of the workforce. At the same time, they were engaging in advocacy with the government’s focal persons who were critical to scaling and sustaining their programs. Perhaps less advocacy was being conducted with communities to raise demand for quality delivery and for the consistent adoption of program messages.

### 4.3. Top-Down and Bottom-Up Approaches to Scaling

The approach in Zambia was mainly a top-down approach to implementing and scaling the Care for the Child’s Healthy Growth and Development program. The Ministry of Health of Zambia worked with UNICEF to implement its flagship CCD program. The Ministry then arranged to identify a volunteer workforce and train them to deliver the program using home visits. This was done before evaluating the effectiveness of the program on parents and children in Zambia [6]. Save the Children’s program, called Prescription to Play, was trialed in its earlier form in several countries and adapted and piloted in Bhutan [8]. Save the Children simultaneously met with government officials to seek their approval and interest. A strictly bottom-up approach taken by many parenting programs starts at the community level by showing the effectiveness of the program, specifically showing changes in stimulating parenting practices and children’s mental development outcomes [2]. It is possible to scale using either approach, for example, Criança Feliz, the CCD program in Brazil, used a top-down approach [7], and others in India and Chile also used a top-down approach [21,22], while Cuna Mas in Peru started with a bottom-up evaluation of the Reach Up program before scaling up [23]. 

There are enablers and challenges to both approaches. The top-down approach ensures that government ministries and other stakeholders are interested in adopting the program. This does not necessarily mean that they will follow through with a policy and budget, but the work of coordinating with the government is assured. Those following the bottom-up approach rarely have easy access to the government. The strength of bottom-up programs is their assurance of having an effective program on a small scale, maybe even using government providers, before they approach the district or national government. Those using the top-down approach rarely have effectiveness data from that site to start, and though they may claim to be “evidence-based”, their evidence is often not strong or from that site.

Clearly, it is useful to work simultaneously from the top-down and the bottom-up. Advocacy to sustain demand and institutionalize the program is needed at the government and the community level. This entails obtaining early buy-in from the government along with workforce development, a policy framework, and financial support to be maintained throughout the transition to scale. It also includes the implementation of home, group, and/or clinic sessions with parents, monitoring their quality, and evaluating their effectiveness. The P2P program in Bhutan comes closest to the ideal of ensuring that they have an effective program within the community while simultaneously working with government ministries at the top.

### 4.4. Limitations

One limitation is the lack of standardized measures for all but the delivery quality tool. The qualitative interviews were guided by specific questions that allowed for content analysis on the issues of interest. Generalizability of the findings is limited by the nature of the workforce, their delivery modality, and the families they contacted. However, programmatic features will be similar to many parenting programs implemented in Africa and Asia. 

## 5. Conclusions and Recommendations for Policy and Investment

This paper presented two case studies of parenting programs developed by organizations in Zambia and Bhutan that were studied by an independent research group. Use of implementation feedback from a research team as well as from the workforce is central to the successful scaling of a program. This study showed different ways that feedback is used and how challenges to scaling were met.

Based on the findings of this research, we offer the following five recommendations for policy and investment:

1. Choice of a promising program and mode of delivery is essential from the start. A situation analysis might indicate that parenting practices are currently not stimulating nor responsive and that the workforce has little experience and expertise in early child development. In this case, a structured prescriptive program is more likely to be effective with both providers and parents in comparison to a flexible light-touch program. Bhutan’s 7-step manual provides a good example of such a program.

2. The policy framework should include options for both home visits and group sessions. Group sessions are more cost-effective, but attendance is a challenge. Home visits give the provider more control but constrain reach. The Zambian program found the need to trial both.

3. The policy framework should include the training and supervision of a sustainable workforce that receives respect and remuneration. A monitoring and information system along with supportive supervision will keep the workforce accountable and maintain quality of delivery. Workload and attrition are two challenges that need to be addressed.

4. Early evidence for effectiveness in improving parenting practices and child development is useful in creating demand and guiding revisions to the program and its quality delivery. An independent research team could conduct regular evaluations using state-of-the-science tools for program evaluations (these are different from tools for demographic surveys).

5. Investment is needed over several years as the program transitions to scale; it should be tied to research evidence that progress is being made in its implementation and outcomes. Data on the costs to initiate and then to sustain the program are needed for the government to make sound decisions.

## Figures and Tables

**Table 1:** Measures; sample sizes; dates when administered.

Measures	Zambia Care for Child’s Healthy Growth and Development (CCD)	Bhutan Prescription to Play (P2P)
1. Observation of delivery		
Date of 1st data collection	December 2021	September 2021
Sample size	N = 60	N = 60
Date of 2nd data collection	August 2023	September 2023
Sample size	N = 60	N = 60
2. Survey of workforce		
Date of data collection	June 2023	August 2023
Sample size	N = 118	N = 150
3. In-depth interviews with workforce		
Date of data collection	June 2023	July 2023
Sample size	N = 18	N = 8
4. Interview with technical resource team		
Date of data collection	August 2023	July 2023
Sample size	N = 2	N = 2

**Table 2:** Quality of delivery observations of Zambia’s Care for Child’s Healthy Growth and Development.

	Round 1 December 2021		Round 3 August 2023	
Quality Item: Specific Activity	% Very Good	Mean ^1^ (SD)	% Very Good	Mean ^1^ (SD)
Ask about homework	21.3	1.5 (0.8)	68.3	2.7 (0.5)
Introduce new play	34.4	1.8 (0.9)	28.3	2.0 (0.8)
Coach responsiveness	19.7	1.4 (0.8)	0.0	1.2 (0.4)
Demonstrate new play	27.9	1.6 (0.9)	18.3	1.7 (0.8)
Give parent opportunity to practice	31.2	1.8 (0.9)	25.0	1.8 (0.8)
Coach new practice	21.3	1.5 (0.8)	3.3	1.3 (0.5)
Explain benefits for child	32.8	1.8 (0.9)	41.7	2.3 (0.7)
Use visual aids	90.2	2.8 (0.5)	56.7	2.5 (0.6)
Offer problem solving	19.7	1.4 (0.8)	13.3	1.5 (0.7)
Review session messages	23.0	1.5 (0.9)	16.7	1.6 (0.8)
Mean score across ten quality items		1.7		1.8
	**Round 1 December 2021**		**Round 3 August 2023**	
**Quality Item: Delivery Skills**	**% Very Good**	Mean * (SD)	**% Very Good**	Mean * (SD)
Well-prepared	55.7	2.5 (0.7)	41.7	2.4 (0.6)
Covered the content	60.7	2.5 (0.7)	40.0	2.3 (0.6)
Lively and engaging	73.8	2.7 (0.6)	48.3	2.4 (0.6)
Acceptance and empathy	70.5	2.7 (0.5)	18.3	2.2 (0.4)
**Parent Engagement**	**% Yes**			
	**Round 1**		**Round 3**	
Did parent complete homework?	31.2		83.3	
Did parent practice with child at visit?	62.3		81.7	
Did parent have own playthings at visit?	42.6		36.7	

**^1^** Specific Activity observations are scored 1 = not observed; 2 = observed, needs improvement; 3 = very good. * Delivery Skills observations are scored 1 = poor; 2 = needs improvement; 3 = very good.

**Table 3:** Quality of delivery observations of Bhutan’s Prescription to Play.

	Round 1 December 2021				Round 2 September 2023			
Quality Item: Specific Activity	%Very Good		Mean ^1^ (SD)		%Very Good		Mean ^1^ (SD)	
Ask about homework	48.3		2.5 (0.6)		85.0		2.8 (0.4)	
Introduce new play	81.7		2.8 (0.4)		93.3		2.9 (0.3)	
Coach responsiveness	16.7		2.0 (0.6)		63.3		2.5 (0.7)	
Demonstrate new play	55.0		2.5 (0.7)		81.7		2.8 (0.4)	
Give parent opportunity to practice	41.7		2.3 (0.7)		73.3		2.7 (0.6)	
Coach new practice	38.3		2.2 (0.8)		66.7		2.5 (0.7)	
Explain benefits for child	63.3		2.6 (0.6)		73.3		2.7 (0.5)	
Use visual aids	23.3		2.0 (0.7)		66.7		2.4 (0.9)	
Offer problem solving	33.3		2.2 (0.6)		71.7		2.6 (0.7)	
Review session messages	63.3		2.5 (0.7)		81.7		2.8 (0.6)	
Mean score across ten quality items			2.4				2.7	
	**Round 1 December 2021**				**Round 3 September 2023**			
**Quality Item: Delivery Skills**	**% Very Good**		Mean * (SD)		**% Very Good**		Mean * (SD)	
Well-prepared	43.3		2.4 (0.5)		86.7		2.9 (0.3)	
Covered the content	68.3		2.7 (0.5)		91.7		2.9 (0.3)	
Lively and engaging	23.3		2.2 (0.4)		86.7		2.9 (0.3)	
Acceptance and empathy	45.0		2.4 (0.6)		86.7		2.9 (0.4)	
	**Round 1 %**				**Round 2 %**			
**Parent Engagement**	**Few**	**Some**		**Most**	**Few**	**Some**		**Most**
Did parent complete homework?	55.8	36.5		7.7	14.04	12.28		73.68
Did parent practice with child during session?	51.9	40.7		7.4	8.93	19.64		71.43
Did parent have own playthings at session?	71.4	28.6		0.0	7.14	57.14		35.71

^1^ Specific Activity observations are scored 1 = not observed; 2 = observed, needs improvement; 3 = very good. * Delivery Skills observations are scored 1 = poor; 2 = needs improvement; 3 = very good.

**Table 4:** Survey results for Zambian and Bhutanese recently trained providers.

Variable	Zambia Care for Child Development	Bhutan Prescription to Play
Demographics		
Current status	Community-based Volunteer	Health Assistant
Age (yr)	37.6	38.9
Sex (% female)	44.9%	49.3%
Education (yr completed)	9.8	13.3
Years of experience in position	7.9	15.7
Workload		
Number of families per week (Za) or past month (Bh)	3.8	19.4 in 2.2 groups
Months of experience delivering parenting program	6.0	7.4
Have responsibilities outside parenting % (hrs/wk)	73.7% (5.0)	100% (37.2)
Feel overworked (%)	22.9%	59.3%
Feel respected and appreciated (%)	100.0%	91.3%
Training and Supervision		
Days trained	6.0	11.4
Refresher (%)	1.7%	86.0%
Supervision in-person past 30 days	87.8%	20.7%
Convened with fellow workers to exchange and support	96.6%	14.7%
Confidence in own delivery ^1^		
Immediate post-training (% 4 or 5 on 5 pt scale)	66.95%	26.7%
Currently (% 4 or 5 on 5 pt scale)	92.37%	86.0%
Self-rated quality of delivery (1 to 10)	9.0	7.8
Caregiver Knowledge of Child Development Inventory		
Ten-item Child development (max twenty)	12.8	13.1
Ten-item Parenting stimulation (max twenty)	12.5	11.2

^1^ Confidence in delivery: 1 = very uncertain; 2 = only a little confident; 3 = somewhat confident; 4 = mostly confident; 5 = very confident.

**Table 5:** Quotes from Zambian in-depth interviews of longitudinal volunteer cohort.

Topic (Valence)	Representative Quotes
Training, best methods used (positive)	We did lots of role-playing then went out to practice on families. They brought dolls and balls for us to practice with.
Materials (positive, negative)	There are seven counselling cards for birth to 24 months of age. But visits are monthly. When we go back a month later [e.g., 7 mo], we use the same card. It’s not boring because the parents can’t understand fast so we have to repeat what we said the month before. Counselling cards—they help us a lot, that’s the backbone; when we reach this age we need this card. The stories and the pictures help us to deliver the lesson. No change, all are in English At least I read but others don’t know how to read English. Nothing was changed. They like songs, in this project there are no songs, we haven’t sung any.
Supervision (negative)	They come, they check our books, records and tell us areas where we should improve. The supervisor needs to be coming again to listen to our complaints. Once since last October, it lasted 40 min. The second of every month we submit a report; do the report on the people that you have visited.Those [supervisors] from government—no, since December they have not come, no. From the health facility, it takes a very short time sometimes they just bring us together they ask us the challenges we have and that’s all. Mostly it is not enough … we should be talking to them for a longer time. They should be coming twice, more especially in the households, not only in the facilities … that means they don’t know what we are doing in the community.
Peer support (positive)	We meet once per month. We sit almost 2, 3 h we go through the reports and talk about other things… we talk to each other the way see that problem, the way anyone can finish that problem in their community, that’s how we help each other. Among ourselves we should be supervising ourselves at times … what I want is when I go to visit [a family], that friend of mine the promoter [CBV] should be maybe like my boss where I am working. That’s what I desire so much.
Refresher (negative)	No refresher. No refresher.
Early child development (positive, negative)	Early child development means teaching the child or making changes in the child from a very young age to the age that maybe they can do things, that a child of that age should be able to do. What it means, teaching a child while they are still young from zero to five years when the brain is growing fast to know things. Play with the child till they go to school. When feeding, hold him like this.
Responsive stimulation (negative)	It is that time when you are talking to the child and the child manages to respond… he manages to follow. When playing …I am giving them a sign and they are signing it back; is the child following me or they are not following me. Responsive stimulation we say that the caregivers need to be responsive to their children helping their children, what their children want, what their children need. Stimulation can mean raising the child to be strong …yes what is meant is that when we are teaching them there, they are playing; their child’s body becomes strong.
Family contact Is it sufficient? (negative)	10 families visited once a month. It is enough. 10 families. Visit twice a month. Parents who are active in my group are 6; the other four you have to remind them. I see 10 families. After 5 visits they graduate and you take another family. You teach them and then go back; if they haven’t learned then you go back again—4 times monthly. See 10 families. I see them once a month its seems that they forget so now I want to … be seeing them twice. In my view once per month is not enough according to the way certain parents are showing, so at least going three times per month. 10 families monthly. Not enough. They forget from one visit to the next. Only 6 of the 10 parents played with their child between the last and current visit.
Challenges in delivering the program (negative)	When it comes to leaving their work they postpone the visit. Or if there is a game in the village, they go to it. During this season they are farming and too busy. Some are slow learners and you pass through the same thing two or three times. Transport and money are lacking for us. We need to have T-shirts that show that you are a promoter from ECD. Solution: The Headman brought parents together and told them ‘these people are a group to teach you the way we can keep our children.’

**Table 6:** Quotes from Bhutanese in-depth interviews of longitudinal health assistant cohort.

Topic (Valence)	Representative Quotes
Training, best methods used (positive)	For our training, our trainers brought in families with children to help with the demonstration and practice. I like the way of teaching … the 7 steps, the demonstration of how to play—mock sessions. Demonstration is better than verbally.
Materials (positive)	The manual is very helpful. If the session is tomorrow, when I flip through the manual, I am able to recollect what was taught, how it is to be delivered. The second version is much better than the first. Songs are good. The caregivers, they have the take home card which has pictures showing them how to play with the child. That reinforces the demonstration we do at the group sessions.
Supervision (negative)	No supervision, though it’s important to have supervision to correct us. Not really. Administrative reporting only. PDSA—they do discuss how the sessions are going, ask about challenges and brainstorm about solutions. We did a lot on how to increase attendance; it’s all good and doable on paper but in reality, it’s still very difficult.
Peer support (positive)	We meet up for 30 min. The frequency and duration of interaction with peers fluctuates. Yes, it is helpful because we get time to discuss ideas and steps about session. We continually talk about problems to come up with solutions, such as attendance and how to encourage parents and fathers to attend. If there were more parents attending, I think we would be more motivated.
Refresher (negative)	No in-person refresher was conducted. Only on zoom and I couldn’t concentrate. I could not attend the refresher course though I was invited, because of staff shortage. I feel like regular refresher courses are needed. Especially since some of the HAs are alone in a particular catchment area and we tend to forget what we learnt in training. I wish to have a refresher course once a year.
Early child development (positive)	Means brain development, early stimulation, health and nutrition, positive parenting and care. Developing the children below age three and providing tips to the parents on developing their children. If we impart more knowledge to them, the child gets rich in knowledge when they grow up. It is about giving proper care and nurture at the younger age and can have positive impact on them.
Responsive stimulation (positive, negative)	Responsive means if a baby is crying, we have to check if maybe it’s because the baby needs diaper change, or medicine, or is hungry, or is uncomfortable. I think responsive is taking the responsibility of responsive parenting, to ensure that they also provide positive care for brain and physical development. And also, to understand cues in children to know their need. How we can recognize and respond appropriately—if a child pulls hair of siblings, how to teach child not to pull, give other options like give clean toys in order to distract … explain and soothe the child. Stimulation means to teach a child to do play and talk activities so that the child is interested to do those. To make the child move, think, speak, etc. So that they interact and respond to us, when we say ‘come here’, ‘catch this’. Responsive means that parents are aware of how to respond to a child, could be when the child is crying. We need to find out why the child is crying, is it because they are hungry, need diaper change or because they are bored. Stimulation means before we play with children, it is about practically showing the way of playing to the children. Responsive means to take a responsibility. Stimulation means that before the parents do activities with their child, we help them learn the right way to do it with their child. I think responsive means to take responsibility to nurture the child through good parenting tips. … responsive stimulation. I have forgotten it. It’s about how to calm the mind of the child and divert the mind of the child.
Family contact. Is it sufficient? (positive, negative)	Groups two times per month. Individuals at clinic 2 times per month. We have regular visits from parents with younger children under 1 year, as they have to come monthly to get the health services… The other parents with older children do not come anyway. Around 50% practice at home. We ask them during the session. We see how well they sing the song that we taught at the previous session. I see 11 families monthly. Bi-weekly sessions are ideal for families but not feasible for us. Only about 50% of the families adopt the new practices. I know that they find it difficult because they tend to forget during our homework review in the next session; like songs they cannot remember or games. Group sessions—about two hours including the waiting. They don’t turn up on time so we sometimes have to wait 30 min to 1 h, and then the sessions take about 1.5 h minimum. Once in a month is not enough. We have discussed to conduct the session twice a month. About 20% of the parents adopt the new play practices. About 50% adopt the communication practices.
Challenges in delivering the program (negative)	Challenges. Shortage of parent attendance, shortage of health worker, heavy workload, and distraction from the children while conducting the session demotivates me. Attendance is a challenge. We call them on the group chat and leave messages one day before about the session. On the day of the session also we call them and remind them to come. Workload: I think we need different staff specifically trained to do the group sessions such as the ECCD [preschool] center facilitators. We may need separate facilitators to do sessions on a regular basis. As HAs with so much work, we cannot always dedicate our time to it. Especially when I am the only one here. At our center, we have no problems with attendance.

**Table 7:** Quotes from the resource team in Zambia.

Topic	Representative Quotes
Improve quality of delivery	“The data that you shared with us … we disseminated to our stakeholders, even the facility supervisor so that they know the findings and the expectations as they monitor the CBVs”. Did not add days to training or a refresher course. “they undergo five to six days training, but that’s a challenge for the government” Supervision mainly administrative: Health facility “workers have extra duties but no extra pay”.
Horizontal reach to service more families in two program districts	Reach: “in the communities we are targeting, our coverage is maybe 20%… it represents a very small portion of that district… But the volunteers work on many projects so they may spread the message farther”. Reduce the number of visits for each household from 10 to 7. Adding group sessions using a group-based manual “communities that are maybe 5 km from the health facility have a designated place where they conduct the activity”. “If we can pitch our advocacy with community leaders [headmen]… we can create demand for the program”. “We want to promote behaviour change… We developed some social behaviour and communication and materials. We’ll launch the materials in two districts … to be shown at health facilities. We also have our banners and jingles on the radio”.
Vertical scale within the health system, e.g., advocacy, training	“We’re training government partners to be able to train CBVs”. Added early child development to nursing curriculum; trained lecturers of nurses: “making sure that the playful parenting components are fully integrated into training for frontline workers”. Continuous need to add more workforce: “Working with volunteers is challenging because of high attrition rates … because they are not getting any payment”. Incentive guidelines from the government are not yet implemented: “we have started to register them”; “they volunteer for different programs… so it has to be harmonized”; “but it’s incredibly complex”. “policy and guidelines for who would hold the contracts, who would pay the money and how it would be budgeted”. Working on a multisectoral policy framework: “right now we don’t have the policy that gives them a mandate to plan for ECD activities within their respective ministries”; “they are constrained with the issues of budget”. Finance depends on showing evidence. “Financing will be linked to ECD output indicators”. “Right now, those budget plans don’t exist”. Activities and financing to be done at the district level, folded into the Ministry of Local Government. “Advocating with the provincial and district heads of department…and parliamentarians … we should also target the Cabinet level”. Advocating at the local level: “involve traditional leadership and communities in decision making. So demand exists there”. “Buy-in is very strong, especially at district [government] level… we need them to take up maintenance both fiscally and technically in order to support and sustain the program. I think they’re forthcoming”. “Lack of information system for ECD… a lack of infrastructure and data collection … for planning and assessment”.

**Table 8:** Quotes from resource team for Bhutan.

Topic	Representative Quotes
Improve quality of delivery	Revision of Manual: “We took all of your advice, and built it into the next phase of the materials. So, on responsive play, for example, we now train them on responsivity… we changed responsive play”. Revised training: We included the changes “in the pre-service training and the refresher training … HAs who will be graduating and those currently in service”. Added our own monitoring: “Initially we were going to use the government monitoring system … but then it got cancelled”; “the university initially agreed… then they realized they couldn’t do it”. “We have actually hired our partner NGO, who we’ve trained on the monitoring tool that you provided which we adopted”. “So, we’ve actually set up something separate for now. And then if the government decides that they’ll take on monitoring, we can bring that back up”.
Horizontal reach into new districts; increasing attendance, demand	Expanded from 5 into 15 new districts at the start of 2023; “trained enough health assistants to cover all the districts”. Discussed poor attendance and how to increase it. “We need to analyze the attendance and the registration details for the remaining 15 districts who started this year …” For example, shorten the length of the session: “we timed the sessions, the actual time that they were spending and realize that if they just were more efficient, it wouldn’t take so long. So, we’ve tried to condense that and just streamline it a little bit more”. “We’ve given them tools [to increase attendance], and we’ll have to see what they come up with—a framework that helps them to be able to see how they can make attendance easier, more attractive, more of a social gathering and more timely and have reminders in place. It’s part of our PDSA cycle”. “Community facilitators who conducted the half day community sensitization workshop could not mobilize most of the caregivers as we expected … scaling up community mobilization, there could be again a bit of trouble there”.
Vertical scale within the health system, e.g., advocacy, workload	University lecturers train new health assistants. “We have integrated [the program] into two modules in the Diploma of Community Health—15 h … We have trained the faculty members who would be responsible for offering those modules”. Continuing to work with “the government information health system to enter our indicators, including sessions conducted and parents attending each session…But I don’t think it will be addressed because it’s a much larger structural challenge”. Former champion in one MoH division “handed the project over to a new team in the non-communicable disease division …our project was added to their existing workload”. The new MoH division “they’re the ones that are concerned about the workload of the health workers”… [providers] “probably don’t have to do all of the 7 steps… we may even reduce the steps and make sessions very short”. Government “they’re waiting to see the research findings” “The difference in the cost that it took us to take this to scale vs. the cost of maintaining it at scale” “The university is an incredible partner for us. I mean, from the start they have been right with us… they were willing to do everything that they could to maintain the project as it was. So, they’ve become very, very good, strong champions”. The Ministry of Education “expressed they would like to see if the ECCD [preschool] facilitators could be trained in this [program] … it’s just not as much rests on one person [HA]”.

## Data Availability

Some anonymized data may be obtained from the authors due ethics restrictions.
